# Vitamin D insufficiency and subclinical atherosclerosis in non-diabetic males living with HIV

**DOI:** 10.7448/IAS.17.1.18945

**Published:** 2014-05-13

**Authors:** Joaquín Portilla, Óscar Moreno-Pérez, Carmen Serna-Candel, Corina Escoín, Rocio Alfayate, Sergio Reus, Esperanza Merino, Vicente Boix, Livia Giner, José Sánchez-Payá, Antonio Picó

**Affiliations:** 1 Infectious Diseases Unit, Alicante University General Hospital-FISABIO, Alicante, Spain; 2 Clinical Medicine Department, Miguel Hernández University, Campus of San Juan, San Juan de Alicante, Alicante, Spain; 3 Endocrinology and Nutrition Department, Alicante University General Hospital-FISABIO, Alicante, Spain; 4 Neurology Department, San Carlos Clinic Hospital, Madrid, Spain; 5 Hormone Laboratory, Alicante University General Hospital-FISABIO, Alicante, Spain; 6 Preventive Medicine Department, Alicante University General Hospital-FISABIO, Alicante, Spain

**Keywords:** atherosclerosis, vitamin D insufficiency, carotid intima media thickness, HIV, antiretroviral treatment, adipokines

## Abstract

**Introduction:**

Vitamin D insufficiency (VDI) has been associated with increased cardiovascular risk in the non-HIV population. This study evaluates the relationship among serum 25-hydroxyvitamin D [25(OH)D] levels, cardiovascular risk factors, adipokines, antiviral therapy (ART) and subclinical atherosclerosis in HIV-infected males.

**Methods:**

A cross-sectional study in ambulatory care was made in non-diabetic patients living with HIV. VDI was defined as 25(OH)D serum levels <75 nmol/L. Fasting lipids, glucose, inflammatory markers (tumour necrosis factor-α, interleukin-6, high-sensitivity C-reactive protein) and endothelial markers (plasminogen activator inhibitor-1, or PAI-I) were measured. The common carotid artery intima-media thickness (C-IMT) was determined. A multivariate logistic regression analysis was made to identify factors associated with the presence of VDI, while multivariate linear regression analysis was used to identify factors associated with common C-IMT.

**Results:**

Eighty-nine patients were included (age 42±8 years), 18.9% were in CDC (US Centers for Disease Control and Prevention) stage C and 75 were on ART. VDI was associated with ART exposure, sedentary lifestyle, higher triglycerides levels and PAI-I. In univariate analysis, VDI was associated with greater common C-IMT. The multivariate linear regression model, adjusted by confounding factors, revealed an independent association between common C-IMT and patient age, time of exposure to protease inhibitors (PIs) and impaired fasting glucose (IFG). In contrast, there were no independent associations between common C-IMT and VDI or inflammatory and endothelial markers.

**Conclusions:**

VDI was not independently associated with subclinical atherosclerosis in non-diabetic males living with HIV. Older age, a longer exposure to PIs, and IFG were independent factors associated with common C-IMT in this population.

## Introduction

Vitamin D insufficiency (VDI) is common in the general population [1]. Its estimated prevalence in people living with HIV is high, ranging from 70.3 to 83.7% [2, 3]. Chronic inflammation due to HIV infection and altered vitamin D (VD) metabolism due to the use of some antiretroviral drugs have been associated with VDI in HIV-infected patients, who are also exposed to common factors related to VDI in the general population [2, 3]. Recently, a EuroSIDA study has pointed to vitamin D deficiency (VDD) as an important co-factor in HIV disease progression and mortality, in the setting of widespread, efficient antiretroviral treatment (ART) [2].

The active metabolite of VD, 1,25 (OH)2 VD, is a steroid hormone that regulates the transcription of approximately 200 genes. Most of these genes are involved in calcium-bone homeostasis, immune function and the cardiovascular or metabolic system [4]. Large epidemiological studies and clinical trials have suggested some association between VDI (25(OH)D blood levels<75 nmol/L) and low bone mineral density, high blood pressure, depression, neurocognitive impairment, type 2 diabetes mellitus, metabolic syndrome, cancer and cardiovascular disease [4, 5].

Several pathogenic mechanisms have been proposed to explain the role of VD levels in cardiovascular disease. VD influences endothelial and smooth muscle cell function by exerting antiproliferative effects on vascular smooth muscle [6] or mediating inflammation by lymphocyte and monocyte macrophage differentiation regulation and release of inflammatory cytokines [7]. VD also modulates the renin-angiotensin-aldosterone system [8]. Different authors have suggested that VD could protect against atherosclerosis, vascular calcification, arterial stiffness and endothelial dysfunction [9, 10]. However, observational and interventional studies have yielded inconsistent results on the association of VDI to subclinical atherosclerosis and carotid intima-media thickness (C-IMT) in the general population and in patients living with HIV [11–13].

The aims of the present study are (1) to evaluate the prevalence of VDI in a population of men living with HIV and its association with cardiovascular risk factors, systemic inflammatory markers, lipodystrophy and ART exposure and (2) to determine whether VDI is associated with C-IMT after adjusting for other traditional and emergent cardiovascular risk factors in the HIV population.

## Material and methods

A cross-sectional observational study was carried out in the Infectious Diseases and Endocrinology Units of a tertiary hospital in Alicante, Spain. The local Ethics Committee approved the study. All men living with HIV belonging to a cohort of 600 patients living with HIV, with regular assessment of endocrine parameters and cardiovascular risk, were proposed to participate in this study if they were ≥18 years of age, ART-naïve or on effective ART (<50 copies RNA/mL), with no changes in the previous six months. With respect to the gender of the study target population, only men were included because at the time of the execution of the study, we lacked a cohort of women with HIV infection with close monitoring of metabolic parameters. Only patients receiving two nucleoside reverse transcriptase inhibitors (NRTIs) with an enhanced protease inhibitor (PIs) or with a non-NRTI [efavirenz (EFV) or nevirapine] that never have been treated with PIs were included. Those with chronic hepatitis C, diabetes mellitus, active AIDS disease, active illegal drug use or psychiatric illness were excluded. No patients were receiving drugs containing calcium or VD. All patients gave written informed consent. The study was conducted in Alicante, Spain, a city on the southeastern Spanish Mediterranean coast (latitude 38°23°N) with more than 320 sunny days a year, between March 2009 and October 2010.

Participants were required to fast for 12 hours prior to the blood sample, which was performed between 8:00 and 9:00 AM. The samples were centrifuged, and serum and plasma were stored at −30°C until determination.

### Outcome variables

#### 25 (OH) VD levels

The serum 25(OH)D levels were determined using a LIAISON^©^ automatic chemiluminescence immunoassay analyser (DiaSorin, Stillwater, MN, USA). Total 25(OH)D: 25-OH-D2+25-OH-D3. Analytical sensitivity: 10 nmol/L. Intra-assay precision: up to 37 nmol/L, coefficient of variance (CV) 4.2%; and 157 nmol/L, CV 3.1%. Inter-assay precision: up to 37 nmol/L, CV 7.7%; and 157 nmol/L, CV 6.4%. The following definitions of VD status were used in this study: 25(OH)D sufficiency: ≥75 nmol/L (VDS); insufficiency: <75 nmol/L; deficiency (VDD): <50 nmol/L.

Secondary hyperparathyroidism was defined as serum 25(OH)D<75 nmol/L and serum parathyroid hormone (PTH)>65 pg/mL (6.9 pmol/L).

#### Subclinical atherosclerosis

Subclinical atherosclerosis was measured by evaluation of C-IMT, measured by ultrasonography (Hitachi EUB-5500HV), with a 7.5 MHz linear probe, using the Mannheim criteria [14]. Both common carotid arteries were evaluated, and IMT was measured automatically in the posterior wall at the end of diastole in a region free of plaque. The volume of interest has a length of 11 cm, adjustable to the visible IMT segment. The mean C-IMT was the mean value of the whole segment. The maximal C-IMT was the maximal value of the region of interest. The measurement was done automatically off-line with the Hitachi team software. Left and right common C-IMTs were evaluated separately, and bilateral C-IMT was obtained by the arithmetic mean of C-IMT of both sides. Results are expressed in millimetres.

### Explanatory variables

As ART therapy can affect VD levels and subclinical atherosclerosis, patients were classified into three groups: (1) those who were naïve to ART; (2) the non-nucleoside group: EFV or nevirapine plus two or three NRTIs; and (3) the PI group: PI plus two or three NRTIs.

Lipid and carbohydrate metabolism variables: fasting glycaemia and basal lipid profile were measured; impaired fasting glucose (IFG) was defined as 5.5 to 7 mmol/L in at least two measurements; glucose was measured using the hexokinase method (Modular auto-analyzer; Roche Diagnostics^®^, Grenzach, Germany), and lipid profile was measured by calorimetric enzymatic techniques (Modular auto-analyzer).

Systemic inflammatory markers: we measured high-sensitivity C-reactive protein (hsCRP) (turbidimetry kinetics; IMMAGE, Beckmann Coulter, Inc., Chasca, MN, USA), plasminogen activator inhibitor-1 (PAI-I), tumour necrosis factor-alpha (TNF-α), soluble forms of TNF-1 and -2 receptors and interleukin-6 (IL-6) (enzyme immunoassay; Quantikine, R&D Systems, Abingdon, UK). Lipodystrophy was determined using a standard questionnaire based on physical examination [15].

### Statistical analysis

Qualitative variables were expressed as relative and absolute frequencies. Parametric variables were expressed as means±standard deviation (SD), nonparametric variables as medians and percentiles 25 to 75. The prevalence of VDI with its 95% confidence interval (95% CI) was calculated. The associations between the different variables and VDI or VD concentrations were calculated using the chi-square test for qualitative variables and the Student *t*-test or Mann-Whitney *U*-test for quantitative variables. The odds ratio (OR) of prevalence was calculated with its 95% CI. A multivariate, unconditional logistic regression analysis was performed to identify factors independently associated with the presence of VDI, using all variables with statistical significance in the univariate analysis, and those considered clinically relevant. For the study of correlations between VD concentrations and quantitative variables, Pearson and Spearman tests were used as appropriate.

We furthermore examined the association between mean C-IMT and the explanatory variables, including VDI. Separate multivariate models were fitted using mean and maximum right and left C-IMT as the dependent variables. Linear regression analysis was used to identify factors independently associated with C-IMT, using all variables yielding statistical significance in the bivariate analysis. In all cases, a *p*-value of <0.05 was considered statistically significant. The SPSS version 19.1 statistical package (SPSS Inc., Chicago, IL, USA) was used throughout.

## Results

The study was offered to 109 men. Nineteen patients refused to give their consent, and a patient was enrolled but did not complete the study, so finally 89 males living with HIV were included in the study, with a mean age of 42±8 years. All participants were Caucasians, and 80.5% were homosexuals. A total of 60.7% were smokers, 32.6% had IFG, 7.9% had high blood pressure and only six patients were taking statins. Socio-demographic, clinical and metabolic variables, lifestyle and ART history are shown in Table 1. There were no differences in clinical characteristics of patients who refused to participate in the study (data not shown). The prevalence of VDI was 80.9% (95% CI: 72 to 89). Twenty-three patients [25.8% (95% CI: 16.7 to 34.9)] had secondary hyperparathyroidism.

**Table 1:** Demographics, clinical and laboratory variables (*n*=89)

Age, years; mean±SD	42±8.2
HIV clinical stage, *n* (%)	
A	53 (59.6)
B	19 (21.3)
C	17 (19.1)
Duration-HIV, years; mean±SD	7.8±5.6
Nadir CD4+, cells/µL; median [P25–P75]	204 [124–287]
Current CD4+, cells/µL; median [P25–P75]	467 [364–677]
Treatment group, *n* (%)	
*Naïve*	14 (15.7)
NNRTI	36 (40.4)
PI	39 (43.8)
Duration exposure, months; mean±SD	
Total ART	67±42
NNRTI	36±27
NRTI	111±90*
PI	55±40
Current exposure to ART (*n*=75),%	
NRTI	
Tenofovir	38
Azidothymidine	17
Lamivudine	35
Emtricitabine	25
Didanosine	8
Abacavir	15
Stavudine	9
NNRTI	
Efavirenz	34
Nevirapine	3
PI	
Lopinavir	23
Atazanavir	10
Fosamprenavir	3
Tipranavir	3
Alcohol consumption (yes), *n* (%)	38 (42.7)
Smoking, *n* (%)	
Never smoker	23 (25.8)
Former smoker	12 (13.5)
Current smoker	54 (60.7)
Sedentary lifestyle (yes), *n* (%)	47 (58)
Body-mass index, kg/m^2^; mean±SD	24.8±3.4
Waist/Hip ratio; mean±SD	0.95±0.1
Lipodistrophy (yes), *n* (%)	27 (30.7)
IFG (yes), *n* (%)	29 (32.6)
Total cholesterol, mmol/L; mean±SD	4.88±1.09
LDL-cholesterol, mmol/L; mean±SD	3.26±1.04
HDL-cholesterol, mmol/L; mean±SD	1.26±0.37
Triglycerides, mmol/L; median [P25–P75]	1.65 [1.1–2.3]
25-(OH)-D, nmol/L; mean±SD	52.2±27

SD, standard deviation; *n*, number of patients; HIV-VL, HIV viral load; ART, antiretroviral treatment; non-NRTI group, current ART with 2 to 3 nucleoside reverse transcriptase inhibitors (NRTIs) plus a non-nucleoside reverse transcriptase inhibitor (non-NRTI) and never received protease inhibitors (PIs); PI group, current ART with 2 to 3 NRTIs plus an enhanced PI; IFG, impaired fasting glucose (plasma glucose: 5.5 to 7 mmol/L); 25-(OH)-D, 25 hydroxy vitamin D;

* sum of exposure.

### VD and antiretroviral therapy

VDI was more prevalent in patients on ART than in naïve patients [OR: 4.3 (95% CI: 1.3 to 15)] (Table 2). Patients in the non-NRTI group had a higher risk of severe VDD, defined as 25(OH)D <25 nmol/L [OR: 6.2 (95% CI: 1.2 to 30.8), *p*=0.02], than those in the PI or naïve groups.

**Table 2:** Vitamin D insufficiency-associated factors: HIV clinical variable and ART

	VDI (<75 nmol/l) *n*=72	No VDI (>75 nmol/l) *n*=17	OR [IC95%]	*p*
Duration-HIV (years); mean±SD	7.7±5.7	7.9±5.4	0.99 [0.9–1.08]	0.8
CD4+ cells/µL; median [P25–P75]	470 [368–689]	442 [359–578]	1.001 [0.9–1.003]	0.3
HIV-VL RNA/mL <39, *n* (%)	53 (73.6)	11 (64.7)	0.65 [0.2–2]	0.4
HIV-VL RNA/mL (naive patients); median [P25–75]	20,100 [2910–44,684]	21,900 [10,000–79,617]	1 [1–1]	0.4
HIV clinical stage; *n* (%):				
A	40 (75)	13 (25)		0.2
B	18 (94)	1 (6)	5.8 [0.7–48.1]	
C	14 (82.3)	3 (17.7)	1.5 [0.37–6.1]	
**Treatment Group;** ***n*** **(%):**				
Naïve	8 (57.1)	6 (42.9)	1	**0.02**
NNRTI	32 (88.8)	4 (11.1)	4.3 [1.3–15]*	
PIs	32 (65.3)	7 (34.7)		
Duration of ART (months); mean±SD	66.8±43.2	69.6±37,4	0.99 [0.98–1.01]	0.8
Duration of PI (months); mean±SD	57±41.8	43.8±26.5	1.01 [0.9–1.03]	0.4
Duration of NNRTI (months); mean±SD	37±27.7	31.1±19.7	1.009 [0.98–1.03]	0.5
Lipodystrophy;% (*n*)	31 (22)	29.4 (5)	1.1 [0.3–3.4]	1

Explanatory variables with statistically significant results in unadjusted analysis and their significance are in bold. VDI, vitamin D insufficiency; SD, standard deviation;%, percentage; *n*, number of patients; HIV-VL, HIV viral load; ART, antiretroviral treatment; non-NRTI group, current ART with 2 to 3 nucleoside reverse transcriptase inhibitors (NRTIs) plus a non-nucleoside reverse transcriptase inhibitor (non-NRTI) and never received protease inhibitors (PIs); PI group, current ART with 2 to 3 NRTIs plus an enhanced PI.

* Naïve patients versus ART patients.

We also analyzed the effect of ART upon the VD levels. Patients receiving any ART combination had lower VD levels than naïve patients (49±26 vs. 71±29 nmol/L, *p*<0.01). We observed differences in comparing treatment with PIs versus non-NRTIs (54±29 vs. 43±21 nmol/L, *p*=0.06), patients receiving EFV versus those with another third agent (43±20 vs. 53±29 nmol/L, *p*=0.09) and patients receiving lopinavir/r versus those without such treatment (58±33 vs. 45±22 nmol/L, *p*=0.05).

### VDI, HIV clinical variables and cardiovascular risk factors

We found a significant association between VDI and a sedentary lifestyle [OR: 6.4 (95% CI: 1.9 to 21.6)], and higher triglycerides concentrations (>1.7 mmol/L) [OR: 9.9 (95% CI: 2.1 to 46.6)]. Although most of the inflammatory biomarkers were elevated in patients with VDI, only PAI-I showed a significant association [OR: 1.22 (95% CI: 1.003 to 1.5)] (Table 3).

**Table 3:** Vitamin D insufficiency-associated factors: cardiovascular risk factors

	VDI (<75 nmol/l)	No VDI (≥75 nmol/l)		
	*n*=72	*n*=17	OR [IC95%]	*p*
Cardiovascular risk factors				
Age (years); mean±SD	42.8±8.4	38.7±7.1	1.07 [0.99–1.15]	0.06
High blood pressure% (*n*)	47.2 (34)	35.3 (6)	1.6 [0.5–4.9]	0.4
**Physical exercise (<3 h/w)**	84.8 (56)	46.7 (7)	6.4 [1.9–21.6]	**0.03**
Smoker (yes)	58.3 (42)	70.6 (12)	0.58 [0.18–1.8]	0.35
SBP (mmHg); mean±SD	127±15	123±12	1.02 [0.98–1.06]	0.3
DBP (mmHg); mean±SD	78±10	72±8	1.06 [0.99–1.1]	0.06
BMI (kg/m^2^); mean±SD	24.9±3.4	23.9±3.3	1.1 [0.9–1.2]	0.2
WHR	0.95±0.07	0.93±0.07	44.5 [0.01–199,546]	0.3
Metabolic Syndrome;% (*n*)	34.7 (25)	11.8 (2)	3.9 [0.8–18.8]	0.08
Metabolic parameters				
Fasting glycaemia (mmol/L); mean±SD	5.37±0.57	5.11±0.37	1.04 [0.9–1.09]	0.2
IFG (yes);% (*n*)	37.5 (27)	11.8 (2)	4.5 [0.96–21.2]	0.057
TC (mmol/L); mean±SD	4.96±1.08	4.47±1.1	1.01 [0.99–1.02]	0.1
LDLc (mmol/L); mean±SD	3.32±1.06	3.1±0.99	1.005 [0.99–1.02]	0.4
HDLc (mmol/L); mean±SD	1.25±0.39	1.34±0.26	0.98 [0.95–1.02]	0.3
**TG (mmol/L); median [P25–P75]**	1.8 [1.39–2.45]	0.99 [0.81–1.42]	1.02 [1.007–1.03]	**0.002**
**TG > 1.7 mmol/L**	56.9 (41)	11.8 (2)	9.9 [2.1–46.6]	**0.001**
Non–HDLc (mmol/L); mean±SD	3.7±1.01	3.13±1.03	1.015 [1.001–1.03]	0.45
Apolipoprotein B (g/L); mean±SD	0.88±0.22	0.77±0.2	12.6 [0.84–191]	0.06
Inflammatory biomarkers				
hsRCP (mg/L); median [P25–P75]	2.9 [0.14–0.57]	1.9 [1.4–3.2]	7.43 [0.5–106.3]	0.1
**PAI-I (pg/L); mean±SD**	9.6±4	7.4±2.4	1.22 [1.003–1.5]	**0.04**
TNF-α (pg/L); median [P25–P75]	0.015 [0.01–0.01]	0.015 [0.01–0.01]	1.07 [0.89–1.28]	0.46
R1 TNF-α (pg/L); median [P25–P75]	0.97 [0.85–1.12]	0.92 [0.77–1.1]	1.001 [0.9–1.003]	0.4
R2 TNF-α (pg/L); median [P25–P75]	1.89 [1.58–2.34]	2.15 [1.77–2.79]	1 [0.9–1.001]	0.2
IL-6 (pg/L); median [P25–P75]	0.003 [0.003–0.003]	0.003 [0.003–0.003]	1.16 [0.87–1.5]	0.32

Explanatory variables with statistically significant results in unadjusted analysis and their significance are in bold. VDI, vitamin D insufficiency; SBP, systolic blood pressure; DBP, diastolic blood pressure; BMI, body mass index; WHR, waist-hip ratio; NRTI, nucleoside reverse transcriptase inhibitor; PI, protease inhibitor; FG, fasting glucose; IFG, impaired fasting glucose (plasma glucose ≥5.55 mmol/L); TG, triglycerides; hsCRP, high-sensitivity C-reactive protein; PAI-I, plasminogen activator inhibitor-1; TNF-α, tumour necrosis factor-alpha; R1 and R2, soluble forms of TNF-α receptors; IL-6, interleukin-6; IMT, intima-media thickness; CC, common carotid.

A multivariate logistic regression analysis that included both variables with significance in the bivariate analysis and relevant clinical variables (age, duration of HIV, CD4+ count and CDC stage) was unable to identify any explanatory variable as an independent factor associated with the presence of VDI, with the exception of triglyceride concentration [OR: 1.02 (95% CI: 1.001 to 1.03, *p*=0.04)].

On analyzing the correlation between VD concentrations and HIV clinical variables or cardiovascular risk factors, the only significant quantitative variables inversely associated with VD concentrations were triglycerides, R: −0.4 (*p*<0.001); non-HDL cholesterol, R: −0.21 (*p*=0.04); hsCRP, R: −0.22 (*p*=0.04); and smoking (year/pack), R: −0.25 (*p*=0.04).

### VDI and subclinical atherosclerosis

**Figure 1:**
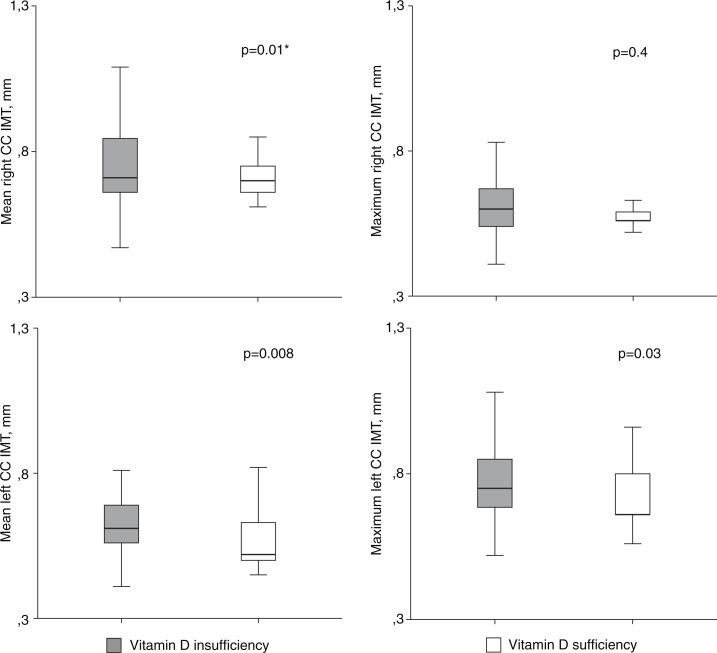
Carotid intima-media thickness by vitamin D category. CC, common carotid; IMT, intima-media thickness. Student *t*-test* or Mann-Whitney U test, as appropriate; *p*, significance. Mean right CC IMT 0.62±0.13 versus 0.56±0.05 mm; maximum right CC IMT 0.71 (0.66 to 0.85) versus 0.7 (0.66 to 0.77) mm; mean left CC IMT 0.61 (0.56 to 0.7) versus 0.52 (0.5 to 0.63) mm; and maximum left CC IMT 0.75 (0.67 to 0.85) versus 0.66 (0.64 to 0.82) mm.

In the univariate analysis, the presence of VDI was associated with greater common C-IMT (Figure 1). Patients with VDI were at a higher risk of having a maximum left C-IMT above median of the study population [OR: 3.2 (95% CI: 1.01 to 0.9), (*p*=0.04)].

There was no correlation between common C-IMT and VD concentrations (as a parametric variable), though patients with VD concentrations below percentile 25 had an increased bilateral maximum common C-IMT (0.77±0.11 vs. 0.71±0.1, *p*=0.06) and bilateral mean common C-IMT (0.63±0.08 vs. 0.56±0.06, *p*=0.09).

The multivariate linear regression model, adjusted by confounding factors, revealed an independent association between patient age, time of exposure to PI and mean/maximum right common C-IMT. In turn, patient age and the presence of IFG were independently associated with mean/maximum left common C-IMT (Table 4). In contrast, there were no independent associations between common C-IMT and VDI or inflammatory and endothelial markers.

**Table 4:** Variables predicting subclinical atherosclerosis in non-diabetic men with HIV infection

	Multiple stepwise regression					
	
	Mean right CC IMT			Maximum right CC IMT		
		
	*B*	*r*^2^	*p*	*B*	*r*^2^	*p*
		**0.45**	**<0.001**		**0.26**	**0.03**
**Age (10 years)**	0.06		**0.004**	0.05		**0.05**
Duration of HIV (years)						
HIV-VL RNA/mL <39 (yes)						
**PIs exposure (years)**	0.02		**0.001**	0.014		**0.03**
NRTI exposure (years)	−0.07		0.2			
NNRTI exposure (years)						
BMI (kg/m^2^)	−0.002		0.7	0.007		0.3
WHR	−0.06		0.8	−0.31		0.4
Lipodystrophy (yes)	−0.02		0.5	0.016		0.7
FG (mmol/L)						
HbA1c (%)	0.07		0.058	0.07		0.14
IFG (yes)	0.03		0.3			
TG (mmol/L)						
hsRCP (mg/dL)	0.04		0.3			
PAI-1 (ng/L)	0.01		0.1	0.01		0.1
IL-6 (pg/L)						
Vitamin D insufficiency (yes)	−0.03		0.4	−0.01		0.8
Constant	0.12		0.6	0.26		0.3
	Mean left CC IMT			Maximum left CC IMT		
		
	*B*	*r* ^2^	*p*	*B*	*r* ^2^	*p*

		**0.44**	**0.003**		**0.48**	**<0.001**
**Age (10 years)**	0.05		**0.01**	0.07		**0.004**
Duration of HIV (years)	−0.02		0.6	−0.006		0.8
HIV-VL RNA/mL <39 cop.(yes)	−0.03		0.4	−0.02		0.5
PIs exposure (years)	0.007		0.2	0.01		0.3
NRTI exposure (years)	0.006		0.3	0.01		0.35
NNRTI exposure (years)				−0.005		0.6
BMI (kg/m^2^)	−0.003		0.5			
WHR	0.04		0.9	−0.32		0.2
Lipodystrophy (yes)	−0.03		0.4	−0.02		0.5
FG (mmol/L)	−0.003		0.2	0.005		0.13
HbA1c (%)	0.05		0.1	0.06		0.1
**IFG (yes)**	0.1		**0.03**	0.13		**0.01**
TG (mmol/L)				0.0001		0.4
hsRCP (mg/L)	−0.00001		0.9			
PAI-1 basal (ng/L)	0.006		0.2	0.009		0.07
IL-6 (pg/L)	0.004		0.2	0.006		0.1
Vitamin D insufficiency (yes)	−0.01		0.8	−0.04		0.39
Constant	0.46		0.12	0.77		0.02

Dependent variables are in bold; explanatory variables with statistically significant results in multiple regression analysis and their significance are in bold. CC, common carotid; IMT, intima-media thickness; B, standardized coefficient; *r*^2^, coefficient of determination; BMI, body mass index; WHR, waist-hip ratio; NRTI, nucleoside reverse transcriptase inhibitor; PI, protease inhibitor; FG, fasting glucose; IFG, impaired fasting glucose (plasma glucose ≥5.55 mmol/L); TG, triglycerides; hsRCP, high-sensitivity reactive C protein; PAI-1, plasminogen activator inhibitor-1; IL-6, interleukin 6.

## Discussion

A high prevalence of VDI (80.9%) has been found in this population of men living with HIV in a sunny Mediterranean city, in coincidence with observations in other studies in HIV populations [2, 3, 16–18]. Twenty-five per cent of the patients with VDI also had elevated PTH concentrations, indicative of severe VD deficiency – this being a situation not previously reported.

In coincidence with other authors, we observed an association between low VD concentrations and antiretroviral therapy, especially EFV [3, 16, 19–22]. EFV induces 24-hydroxylase, an enzyme of the CYP-P450 complex which hydrolyzes 25(OH)D to the inactive metabolite of VD, 24,25(OH)_2_D, reducing the concentrations of the active metabolite of VD [23]. Other variables of HIV infection, such as the nadir or basal CD4+ counts, HIV viral load, previous AIDS, clinical stage, lipodystrophy or years with HIV infection, were not associated with VDI, as was noted in the EuroSIDA study [2].

A significant association between VDI and C-IMT was observed in the univariate analysis. But multivariate analysis was unable to confirm such an independent association between VDI and an increase in common C-IMT. Only older age, IFG and treatment with PI were independently associated with subclinical atherosclerosis. In contrast to our results, two cross-sectional studies have reported an independent association between VDI and C-IMT in people living with HIV in the multivariate regression analysis [12, 13]. The clinical characteristics of the population, the study design, the methodology and the explanatory variables included in the regression analysis could explain differences among studies.

Observational and interventional studies yield inconsistent data on the association between VD and subclinical atherosclerosis markers such as C-IMT and cardiovascular disease in the general population [10, 24]. Two randomized, double-blind, placebo-controlled trials have shown no improvement in endothelial function, arterial stiffness or inflammation markers after VD supplementation in the general population [25] and in people living with HIV [26]. In the EuroSIDA cohort study, involving 1985 patients with five years of follow-up, lower VD concentrations were not associated with increasing incidences of cardiovascular disease [2].

People living with HIV have greater C-IMT [27] and an increased risk of cardiovascular disease and myocardial infarction compared with matched non-HIV controls [28]. The possible relationship between ART and cardiovascular disease is controversial [29–32]. Our study found an independent association between common C-IMT and patient age, IFG and time of exposure to PI (increase of 0.1 mm in C-IMT if IFG exists, or for every five years of PI therapy). In the general population, the adjusted relative risk of stroke is increased by 18% per 0.1 mm increase in C-IMT, and the risk of myocardial infarction is increased by 15% [33, 34]. Previous studies have described an accelerated progression of atherosclerosis in patients living with HIV with cumulative exposure to PI therapy [30, 35, 36]. In subjects without HIV and without diabetes, the presence of glucose intolerance (IFG or impaired glucose tolerance) is associated with a significant increase in C-IMT [37, 38]. This is the first time that such an association is reported in non-diabetic men living with HIV.

Our study has some limitations. Its cross-sectional, observational design precludes definitive conclusions regarding the causal relationships, only associations. Also, we have not included a control group without HIV infection and we have not evaluated solar exposure, seasonal factor or VD intake from food sources. The use of specific markers to monitor chronic inflammation, such as alpha-1 acid glycoprotein, increases in which are more associated with early and late convalescence, may change the correlation between VD insufficiency and inflammatory markers. On the other hand, the study also has several unique strengths: we measured C-IMT, which is a strong and sensitive surrogate marker of the earliest changes of atherosclerosis, and we made a careful assessment of several potential confounders of the C-IMT and VD status relationship, adjusting for traditional and emergent cardiovascular risk factors.

## Conclusions

In summary, we have observed a high prevalence of VDI in a population of men living with HIV in a sunny Mediterranean city. Older age, a longer exposure to PIs, and IFG are independent factors associated with subclinical atherosclerosis measured by C-IMT in males living with HIV and with low cardiovascular risk. Therefore, our data suggest that patients living with HIV who are exposed to PI, especially older individuals or those with impaired glucose metabolism, are at special atherosclerosis risk.

VDI is associated with other cardiovascular risk factors and could behave as a surrogate marker of subclinical atherosclerosis in non-diabetic males living with HIV. Hence, the role of VD in atherosclerosis, if any, remains elusive and poorly defined, and preventive interventions should focus on traditional cardiovascular risk factors. Probably only prospective studies with clinical endpoints will give a definitive answer.
